# Conducting Composite Material Based on Chitosan and Single-Wall Carbon Nanotubes for Cellular Technologies

**DOI:** 10.3390/polym14163287

**Published:** 2022-08-12

**Authors:** Vera Vladimirovna Kodolova-Chukhontseva, Mikhail Alexandrovich Shishov, Konstantin Andreevich Kolbe, Natalia Vladimirovna Smirnova, Irina Petrovna Dobrovol’skaya, Elena Nikolaevna Dresvyanina, Sergei Gennadievich Bystrov, Nadezda Semenovna Terebova, Almaz Maratovich Kamalov, Anna Ericovna Bursian, Elena Mikhailovna Ivan’kova, Vladimir Evgenievich Yudin

**Affiliations:** 1 Research Laboratory “Polymer Materials for Tissue Engineering and Transplantology”, Institute of Biomedical Systems and Biotechnology, Peter the Great St. Petersburg Polytechnic University, Polytechnicheskaya Street, 29, 195251 Saint-Petersburg, Russia; 2 Laboratory No 8—Mechanics of Polymers and Composite, Institute of Macromolecular Compounds Russian Academy of Science, V.O., Bolshoy pr. 31, 199004 Saint-Petersburg, Russia; 3 Institute of Textile and Fashion, Saint Petersburg State University of Industrial Technologies and Design, Bolshaya Morskaya Street, 18, 191186 Saint-Petersburg, Russia; 4 Department of Physics and Surface Chemistry, Udmurt Federal Research Center UB RAS, Tatiana Baramzina Str., 34, 426067 Izhevsk, Russia

**Keywords:** chitosan, single-walled carbon nanotubes, film scaffolds, morphology, mechanical properties, conductivity, electrical stimulation, human dermal fibroblasts

## Abstract

Biocompatible electrically conducting chitosan-based films filled with single-wall carbon nanotubes were obtained. Atomic force microscopic studies of the free surface topography revealed a change in the morphology of chitosan films filled with single-wall carbon nanotubes. Introducing 0.5 wt.% of single-wall carbon nanotubes into chitosan results in an increase in tensile strength of the films (up to ~180 MPa); the tensile strain values also rise up to ~60%. It was demonstrated that chitosan films containing 0.1–3.0 wt.% of single-wall carbon nanotubes have higher conductivity (10 S/m) than pure chitosan films (10^−11^ S/m). The investigation of electrical stimulation of human dermal fibroblasts on chitosan/single-wall carbon nanotubes film scaffolds showed that the biological effect of cell electrical stimulation depends on the content of single-walled carbon nanotubes in the chitosan matrix.

## 1. Introduction

In recent years, polymers have been widely used in cellular technologies, regenerative medicine, manufacturing of medical devices for diagnostics, and treatment of various diseases. Implementation of these technologies requires new materials, which should be non-toxic for human cells and possess biocompatibility and conducting properties.

It has been demonstrated that electrical stimulation is especially efficient in regeneration of tissues of central and peripheral nervous systems [1]. This method can be also used for treatment of a damaged myocardium [2], regeneration of skeletal muscles [3], acceleration of wound healing [4], and bone tissue reconstruction [5].

Electric fields exert an influence on intracellular biochemical processes and determine parameters of intercellular medium. These parameters, in turn, control the main cellular processes: adhesion, migration, proliferation, differentiation, and cell apoptosis. At the tissue level, electrical stimulation influences the rate and efficiency of cell communication and regenerative processes.

To study the influence of the electric field on cellular processes, to provide efficient stimulation of these processes, and to control them, it is necessary to prepare a scaffold that will be able to transmit electrical signals of various intensities and frequencies to the cellular structures. The possibility of non-invasive cellular regulation increases efficiency of regenerative technologies [6].

An example of electrical regulation of the regenerative processes is maintaining a constant electric potential between outer and inner skin layers, which plays a certain role in preservation of homeostasis and regeneration of damaged skin. Long-term disruption of the electric potential between outer and inner layers of skin may result in the appearance of chronic wounds [7,8].

The processes of regeneration of wounds and soft tissues involve dermal fibroblasts; therefore, fibroblasts are frequently used as the model cells in development and optimization of biocompatible conducting materials, biomedical devices, and electrostimulation protocols. Wounds of various etiologies are treated with atraumatic wound dressings, which undergo resorption under the action of biological media and stimulate cellular processes that accompany regeneration of the wound surface [9,10].

The second important direction in the application of bioresorbable electrically conducting films is their use as the main components of conduits, the constructions intended for stimulation of regenerative processes in peripheral nerves [1,11]. Therefore, development of conducting films based on resorbable polymers is an actual problem in tissue engineering.

The composite materials used in tissue engineering should reproduce the properties of the surrounding native tissues as accurately as possible, i.e., should mimic the properties of natural tissues. These polymers include natural macromolecules: chitosan, polylactide, gelatin, and alginate. To impart conductivity to a polymer, carbon materials or conducting polymers are introduced into a scaffold. In particular, carbon nanotubes are used for biomedical purposes, because they combine high electrical conductivity and high specific surface area [12].

In order to provide good biocompatibility, it is essential that the electrical characteristics of a material are close to the electrical characteristics of living cells and tissues. Materials with high conductivity may cause cell death when the value of the applied current strength exceeds cell survival threshold, whereas materials characterized by insufficient conductivity may cause overheating upon applying voltage. Overheating, in turn, is also harmful, because it induces denaturation of proteins and formation of toxic products in a cultural medium in vitro or in tissue liquids in vivo [13].

The basic factor that should be taken into consideration in the development of conductive materials that have contact with living cells and tissues is a mechanism of combined action of the electric signals caused by electron conductivity of the materials and the signals of ionic conductivity that are received by cells. In addition to the type of conductivity of the material and electrical properties of the scaffold, biological effect of electrostimulation on this scaffold depends on electrochemical processes in the near-surface layer of the scaffold material. Understanding of these processes allows one to select the parameters of currents that act upon a cell membrane during electrostimulation.

In this work, chitosan-based composites modified with single-wall carbon nanotubes (SWCNT) were used as biocompatible conducting scaffolds. Chitosan, a derivative of polysaccharide chitin, possesses a set of useful biological properties, such as biocompatibility, biological activity, bioresorbability, antibacterial, wound healing, and hemostatic properties. In addition, it has been reported in several papers [14,15,16,17] that neither chitosan nor the products of its biodegradation show cytotoxicity; thus, chitosan can be recommended for application in skin regenerative technologies.

Carbon nanoparticles of various shapes have been widely used in medical purpose materials [13,18,19]. It is expected that introduction of single-wall carbon nanotubes into the chitosan matrix will provide the necessary level of conductivity; there is also a high probability that the presence of SWCNT will improve mechanical properties of the composite film.

Previously, in [20], a method for obtaining biocompatible electrically conducting chitosan-based films was described and structural studies were given. This paper presents a detailed study of the electrically conductive and capacitive properties, morphology and chemical composition of the surface, mechanical characteristics of electrically conducting chitosan-based composite materials, as well as a study of the effect of electrical stimulation on the adhesion and proliferative activity of human dermal fibroblasts using the prepared chitosan/SWCNT matrix film.

The aim of the present work is a preparation of the composite films based on chitosan and SWCNT with optimal mimicry and electrically conductive properties for in vitro studies of the effect of an electric field on the proliferative activity of cells. This research is needed for the further development of biomedical devices based on the obtained electroactive biocompatible composite materials.

## 2. Materials and Methods

Composite films were prepared on the basis of chitosan with molecular mass Mm = (1.64–2.1) × 10^5^ and deacetylation degree DD = 92% purchased from Biolog Heppe GmbH (Germany).

The films were obtained from the mixture of the 4% solution of chitosan in 2% acetic acid and an aqueous dispersion of SWCNT (Carbon Chg, Chernogolovka, Russia). The diameter of SWCNT was equal to 1.4 ± 0.3 nm; their length was 1–5 µm [21].

Before preparation of the working solutions, the aqueous dispersion containing SWCNT was subjected to ultrasound treatment with an IL10-0.63 dispersant (Russia) for 15 min (25 kHz, 630 W).

Chitosan powder was gradually introduced into the aqueous dispersion; the mixture was stirred for 1 h until the chitosan swelled and partially dissolved. Next, acetic acid was gradually added to the mixture until its concentration in solution became equal to 2%. The suspension was stirred for 6 h, then filtered and deaerated in a vacuum chamber for 24 h at a pressure of 10 kPa. The SWCNT content was equal to 0.1–3 wt.% with respect to chitosan.

**Figure 1:**
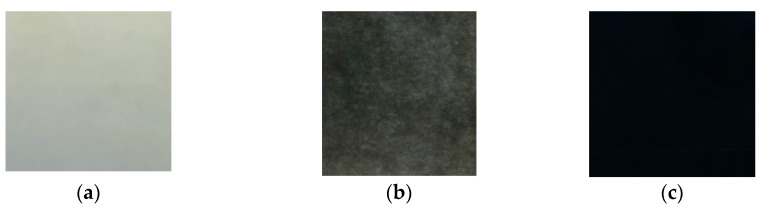
Optical images of chitosan-based films containing 0, 0.5, and 3 wt.% of SWCNT ((**a**), (**b**), and (**c**), respectively).

The chitosan–SWCNT films were produced by extruding the mixture of chitosan and SWCNT through a slit die onto a glass substrate, followed by heating at 50 °C for 1 h. The films on glass substrate were deaerated in a vacuum chamber for 24 h at 10 kPa and dried in air at room temperature for 24 h. The obtained films were exposed to 10% aqueous solution containing NaOH and C2H5OH (1:1) for 10 min, then washed with distilled water and dried in air [22,23]. The films cast from the obtained solutions had a homogeneous appearance and a uniform black coloring (Figure 1).

The surface of the composite films (free surface) was studied by atomic force microscopy (AFM) with the aid of a SOLVER PRO scanning probe microscope in the height and lateral force contact modes. The AFM images were processed using a special software, and, for each sample, the statistical parameter (the maximum ten-point height difference RZ) and the measurement error for each point (the standard deviation) were calculated.

The X-ray photoelectron spectroscopy studies were conducted using a magnetic spectrometer (resolution: 10–4, spectrometer luminosity: 0.085% at excitation of the AlKα-line 1486.5 eV [24]). The C1s photoelectron spectra of the internal levels of chitosan and the composite films were obtained.

The structure of nanocomposite chitosan film in the basic form was investigated by scanning electron microscopy (SUPRA-55VP instrument, Carl Zeiss, Germany).

Mechanical properties of the samples were studied with an Instron 5943 universal testing machine; the base length was 10 mm, and the sample extension rate was 10 mm/min. Before measurements, the tested films were kept in a desiccator at a relative air humidity of 66% for not less than 24 h.

Mechanical properties of the prepared composite films in physiological solution were investigated using an Instron ElectroPulse E1000 setup equipped with a Biss Ligagen bioreactor; the base length was 10 mm, and the temperature in the bioreactor was 37 °C.

Electronic absorption spectra of solutions of MTT and formazan in dimethylsulfoxide (DMSO) were taken with an SF-256 spectrophotometer (LOMO Photonika, Saint Peterburg, Russia) in the wavelength range from 250 to 900 nm at room temperature.

The in vitro studies were carried out with the use of the culture of human dermal fibroblasts obtained from the Collection of Cell Cultures of the Institute of Cytology, RAS (Saint Petersburg, Russia). The cells were cultivated in DMEM nutrient medium (PanEco, Moscow, Russia) containing 1% of L-glutamine, 1% of antibiotics (penicillin 100 units/mL, streptomycin 100 µg/mL), 1% of fungizone (amphotericin B, 25 µg/mL), and 12% of fetal bovine serum (Gibco, New York, NY, USA). The cells were cultivated in a CO_2_ incubator at 37 °C; the CO_2_ concentration was 5%.

The prepared film samples were sterilized in an autoclave for 40 min (121 °C, 1.5 atm). The circular fragments of the films were placed in the wells of a 24-well cultural plate, and the suspension of cells (25 × 10^3^) in the complete cultural medium was added to each well. The cells grown on culture plastic supports were used as a control.

The MTT tests were carried out after cultivation of cells for 1 and 4 days; 100 µL of MTT working solution was added into each well, and the samples were incubated for 2 h. The developed formazan crystals were extracted by adding 1 mL DMSO into each well. Optical density of the resulting solution was measured with a SPECTROstar Nano spectrophotometer at the wavelength of 570 nm. Optical density of the solution correlates with the amount of the present viable cells.

## 3. Study of the Influence of Electrostimulation on Cell Vital Activity

Design of the cell used in the studies of influence of electrical stimulation on cell vital activity is described in [13,20]. The cell consists of a Teflon bath and frame as well as platinum wire electrodes that are 1 mm in diameter. The studied film sample was placed onto the cell bottom and pressed with the frame. The distance between parallel electrodes was 9 mm, and the electrode length was 2.5 cm. The leak-tight cell provided reliable fixation of a film and the necessary gas exchange. Electrical stimulation of cells was performed using an ELINS Potentiostat-Galvanostat with an operating current range of 10^−9^ to 2.0 A and an operating voltage range from 8 × 10^−6^ to 15 V; the rate of signal registration was 1580 points/s.

The influence of electric field on adhesion and proliferative activity of human dermal fibroblasts was studied in the cell described above. The suspension of fibroblasts (25 × 10^3^) in the complete cultural medium was applied onto the surface the preliminary sterilized film. The control standard vial containing a similar number of dermal cells was used for comparison. The electric cell and the control vial were placed into a CO_2_ incubator for 24 h. On the second day, electrical stimulation of cells was performed for 4 h by continuous delivery of the U-shaped signal (amplitude: ±100 mV, period: 60 s) to the film.

One hour after completion of electrical stimulation, fibroblasts from the cell and the reference vial were transferred to other vials (surface area 12.5 cm^2^) and kept in the CO_2_ incubator until a monolayer was formed on the surface.

When the monolayer was formed, the cells were removed from vials and placed onto an 8-well plate of an RTCA iCELLIgence analyzer (5 × 10^3^ cells per well). The cell growth rate was monitored in real time for 7 days by measuring electric impedance of the sensors located at the bottom of the wells with fibroblasts. The measured value of electric impedance corresponds to the value of cell index (CI) [25].

When no cells are present, or they are not attached to the film electrode, the CI value is equal to the background value (which is close to zero). The CI values increase as cells become attached to film electrodes.

In these experiments, time dependences of cell indices of the stimulated and reference cells were obtained [26].

## 4. Results

**Figure 2:**
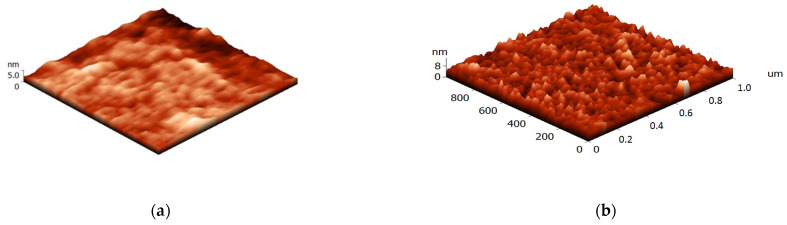

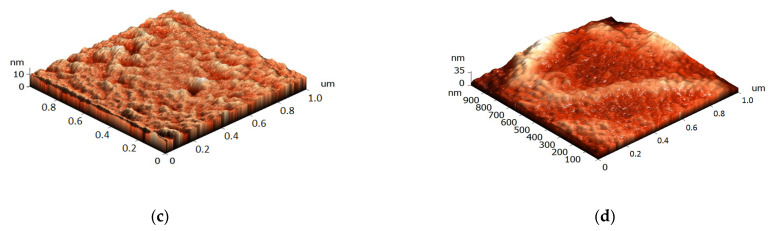
Three-dimensional images of the surface of the chitosan film (**a**) and composite films containing 0.5 (**b**), 1.0 (**c**), 3.0 (**d**) wt.% of SWCNT. Size of the studied surfaces: 1 × 1 µm.

**Figure 3:**
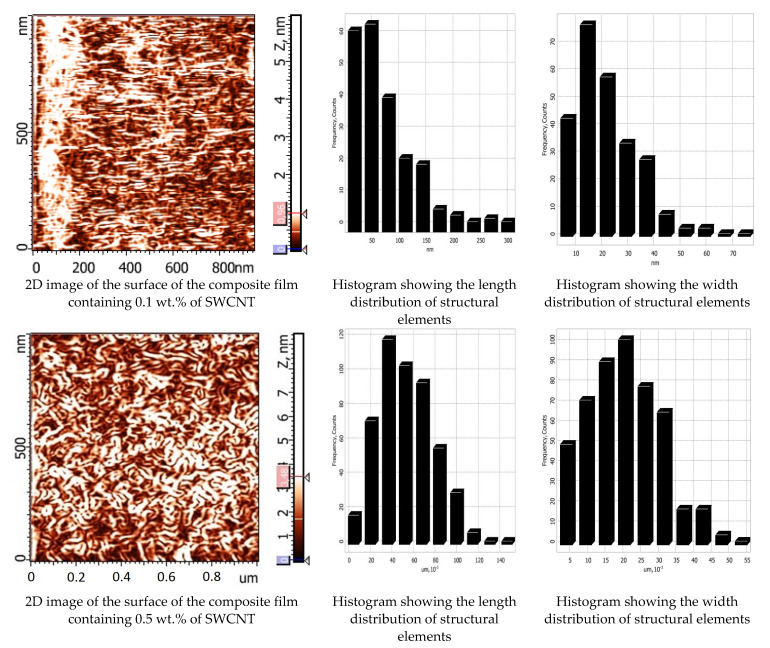

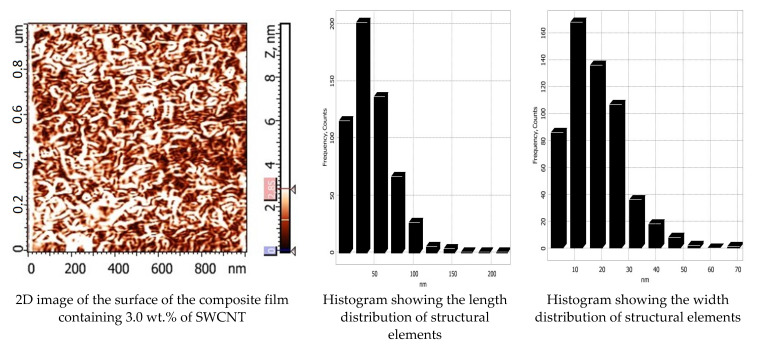
Two-dimensional images of surfaces of the composite films containing 0.1, 0.5, and 3.0 wt.% of SWCNT; histograms of length and width distributions of the structural elements.

The AFM studies of free surface topography demonstrated that morphology of the chitosan films filled with carbon nanotubes (Figure 2) differs from that of the pure chitosan sample. The two-dimensional images of the surfaces of the composite films and histograms showing size distributions of the particles on them are presented in Figure 3. The obtained images of the film surfaces were used to calculate statistical parameters of their topography: the arithmetic average roughness (Ra) and the maximum 10-point height difference (RZ);

It is seen that carbon nanotubes are uniformly distributed in the film plane; the roughness increases with increasing SWCNT concentration from 0.1 to 3.0 wt.% (Figure 2). Histograms of distribution of structural elements for the composite films filled with 0.1–3.0 wt.% of SWCNT are almost similar, despite a considerable difference in amounts of the filler. When 0.1 wt.% of SWCNT is introduced, morphology of the composite film remains unchanged as compared to that of the pure chitosan film; carbon nanotubes are localized between rigid chitosan macromolecules (Figure 3).

**Figure 4:**
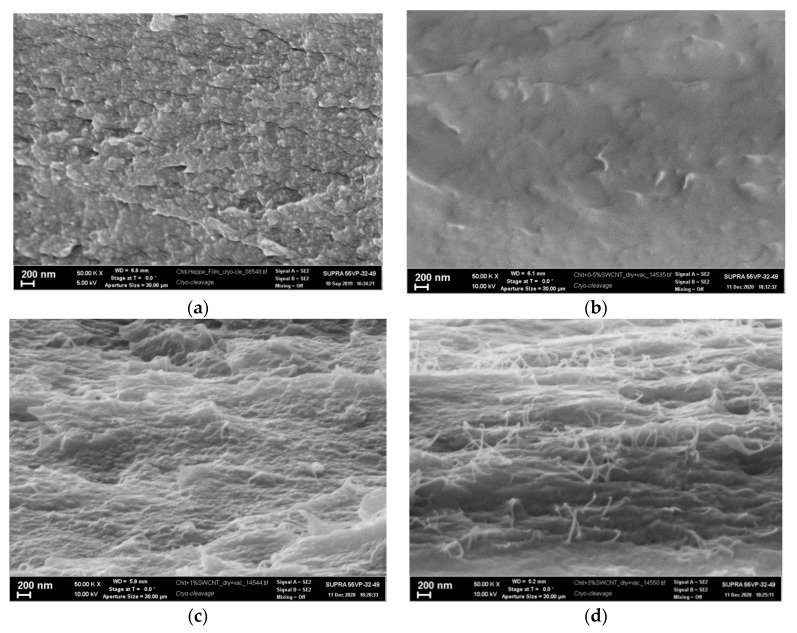
Microphotographs of the fracture surfaces obtained in liquid nitrogen: chitosan film (**a**), composite films containing 0.5 wt.% (**b**), 1 wt.% (**c**), and 3 wt.% of SWCNT (**d**).

As indicated in Figure 3, chitosan macromolecules in the composite film containing 0.5 wt.% of carbon nanotubes are densely packed. These data are confirmed by the results of the SEM studies of the structure of the composite films (Figure 4). The dimension range of the observed structural elements (length: 10–140 nm, width: 5–50 nm) is narrower than that found for similar films containing 0.1 and 3.0 wt.% of SWCNT (length: 25–300 nm, width: 10–80 nm).

Analysis of microphotographs (Figure 4) of the chitosan film and composite films containing SWCNT reveals that introducing 0.5 wt.% of SWCNT into chitosan results in the formation of a denser and homogeneous film. When the content of SWCNT increases up to 1.0 wt.%, individual carbon nanotubes are seen on the fracture surface (Figure 4c). The amount of nanotubes of the surface increases upon introduction of 3 wt.% of SWCNT (Figure 4d).

**Figure 5:**
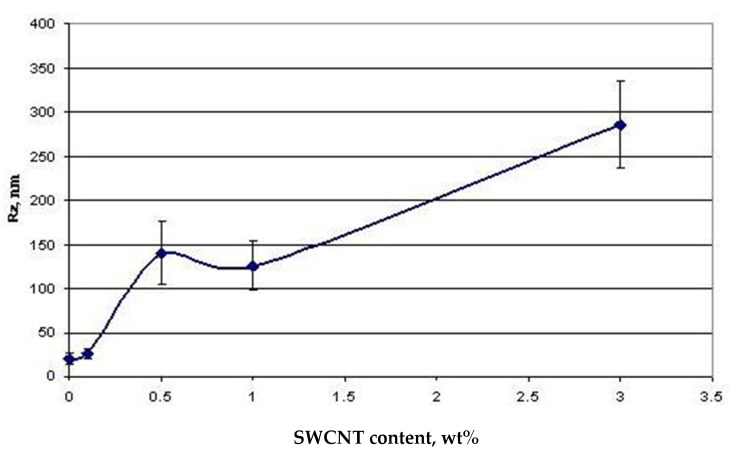
Dependence of the maximum height difference (R_Z_) on the surface of the composite films on SWCNT concentration.

Figure 5 presents the dependence of the maximum height difference on the samples’ surfaces on the SWCNT concentration. It is seen that surface roughness of the composite film containing 3.0 wt.% of SWCNT is 10 times higher than that of the pure chitosan film. The SEM data (Figure 4) imply that saturation of the chitosan scaffold with carbon nanotubes (up to 3 wt.% with respect to chitosan) leads to formation of a network of carbon nanotubes, which are located both in the film plane and perpendicularly to this plane.

Chemical compositions of surface and near-surface layers of chitosan and composite films were studied by X-ray photoelectron spectroscopy (XPS).

**Figure 6:**
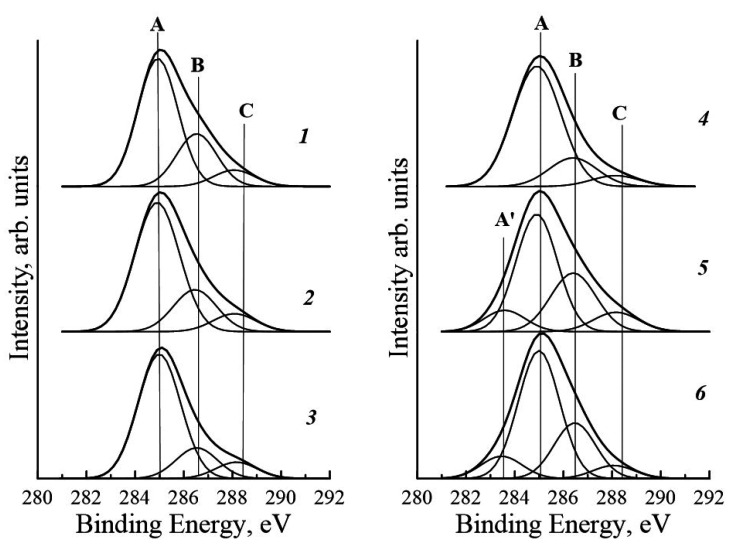
X-ray photoelectron spectra of internal layers C1s of chitosan (**1**) and composite films containing 0.1 (**2**), 0.5 (**3**), 1.0 (**4**), 2.5 (**5**), and 3.0 (**6**) wt.% of SWCNT. Peak A—C-H (285.0 ± 0.2 eV); peak B—C-C (286.4 ± 0.2 eV); peak C—C-O-H, C-O-O-H (288.4 ± 0.2 eV), 283.42 ± 0.2 eV (peak A’).

Figure 6 presents XPS spectra of C1s levels of chitosan and composite films. It is seen that the C1s spectral line of the pure chitosan film consists of three components: peak A—C-H (285.0 ± 0.2 eV); peak B—C-C (286.4 ± 0.2 eV); peak C—C-O-H, C-O-O-H (288.4 ± 0.2 eV).

The C1s XPS spectra of the composite films containing 0.1, 0.5, and 1.0 wt.% of SWCNT virtually coincide with the spectrum of the chitosan film. In the spectra of the samples containing 2.5 and 3.0 wt.% of SWCNT (curves 5, 6), the maximum near 283.42 ± 0.2 eV (peak A’) appears; this peak is attributed to bonds between sp-hybridized carbon atoms. This result indicates the presence of SWCNT on the surface of composite films, which leads to an increase in surface conductivity of the samples.

Note that, according to the data reported in [27], the presence of both types of conductivity (ionic and electronic) in a medium is optimal for cellular processes (adhesion and proliferation). When SWCNT are in the surface layer of a composite material (samples containing 2–3 wt.% of nanotubes), the electronic component of conductivity in the surface layer will be higher as compared to that of the samples containing 0.1–2 wt.% of SWCNT.

Table 1 gives mechanical characteristics of the pure chitosan film and the composite films containing 0.1–3.0 wt.% of SWCNT. It is apparent that filling chitosan with carbon nanotubes leads to an increase in tensile strength (by 27–45%) and tensile strain of the chitosan-based films (by 28–57%). Good mechanical parameters of the composite film containing 0.5 wt.% of SWCNT are related to high dispergation of nanotubes in the bulk of the chitosan scaffold and denser packing of chitosan macromolecules (as compared with that in pure chitosan); this is confirmed by the data of X-ray diffraction analysis [20], scanning electron microscopy, and atomic force microscopy.

Polymeric scaffolds developed for cellular technologies operate in liquid biologically active environments. Therefore, mechanical characteristics of the prepared composite films were investigated in physiological solution at T= 37 °C. Table 2 presents mechanical characteristics of the chitosan film and the composite film containing 3.0 wt.% SWCNT, which were determined in physiological solution. It was shown that introducing SWCNT results in an increase in the tensile strain and tensile strength values of the composite films in physiological solution in comparison with those of the pristine chitosan film.

**Figure 7:**
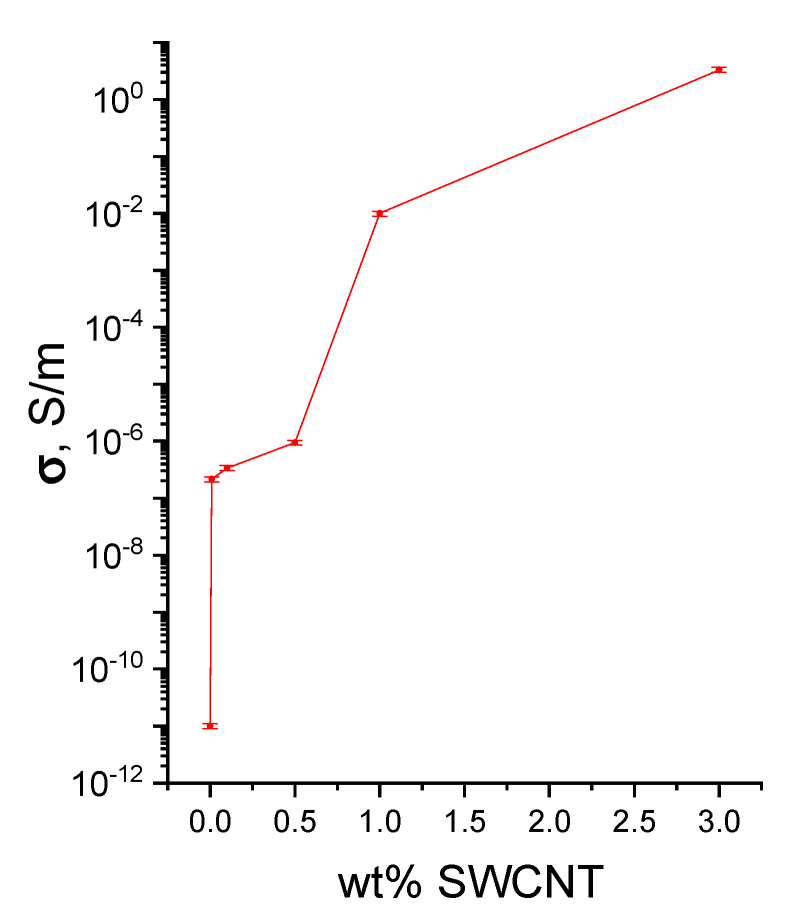
Dependence of specific conductivity of the chitosan/SWCNT composite films on the content of carbon nanotubes.

Figure 7 displays the dependence of specific conductivity of the chitosan-based films on the SWCNT content.

It is evident from the plot that an increase in the SWCNT percentage from 0.5 to 1.0 wt.% leads to an increase in conductivity of the composite film (from 10^−6^ to 10^−2^ S/m). This sharp rise in the material conductivity from a relatively small increase in filler content indicates the formation of a conducting cluster consisting of SWCNT.

The increase in the filler content up to 3 wt.% results in further growth of conductivity; however, the growth rate decreases slightly.

The scaffolds used in cellular technologies should possess a set of electrical properties that facilitate the interaction between cells and the external liquid matrix and the cell–cell interactions. These interactions determine the processes of adhesion, proliferation, and differentiation of cells.

**Figure 8:**
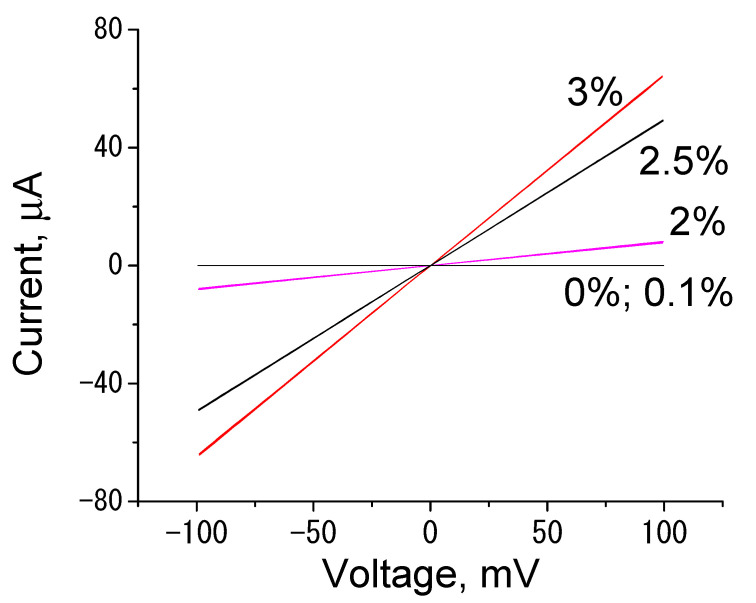
Voltage−current relationships of dry chitosan−based films containing 0, 0.1, 2.0, 2.5, and 3.0 wt.% of SWCNT.

Figure 8 shows voltage–current relationships of dry composite films containing 0–3.0 wt.% SWCNT. It is seen that the voltage–current characteristics are linear, which indicates predominantly electronic conductivity in dry films. Conductivity of the films increases with increasing SWCNT concentration. The conductivity of the film containing 2.5 wt.% of SWCNT is more than six times higher than that of the film containing 2.0 wt.% of SWCNT. This great difference in conductivity between the samples with close contents of the conducting component indicates the formation of the developed percolation conducting cluster.

**Figure 9:**
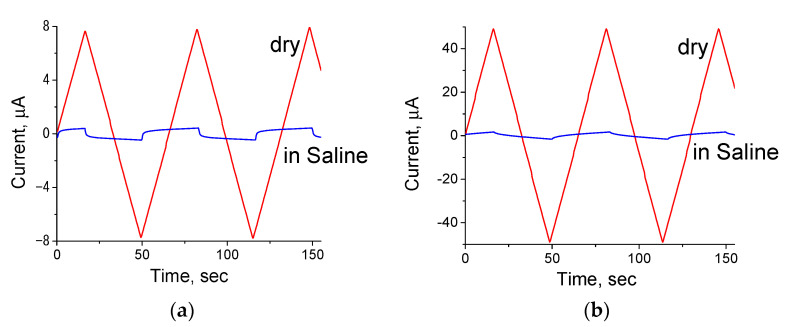
Current sweeps for the chitosan/SWCNT composite films measured in the dry state (red) and in physiological solution (blue). The SWCNT contents: 2.0 wt.% (**a**), 2.5 wt.% (**b**).

After exposure of the films to physiological solution for 1 min, a sharp change in their conductivity is observed [28,29]. At similar voltages, the currents in the wet scaffold are dozens of times lower than those in dry scaffolds, and the voltage–current relationships are curves reaching a plateau (Figure 9). This is related to the fact that the hydrophilic chitosan scaffold is saturated with electrolyte ions; in addition, chitosan contains ionogenic NH^3+^ groups. In physiological solution, these ionogenic groups become hydrated, and chitosan behaves as an ionic conductor.

Figure 9 illustrates the sweeps of currents for the samples containing 2.0 and 2.5 wt.% of SWCNT (dry films and the films in physiological solution) obtained upon application of saw-tooth-like potential ±100 mV, whose polarity was changed every 30 s. The current in the dry scaffold is considerably higher than that in the scaffold containing physiological solution. The current in the dry scaffold synchronously changes with a change in voltage; the currents in physiological solution are inertial, and a delay is observed between the applied voltage and the current.

**Figure 10:**
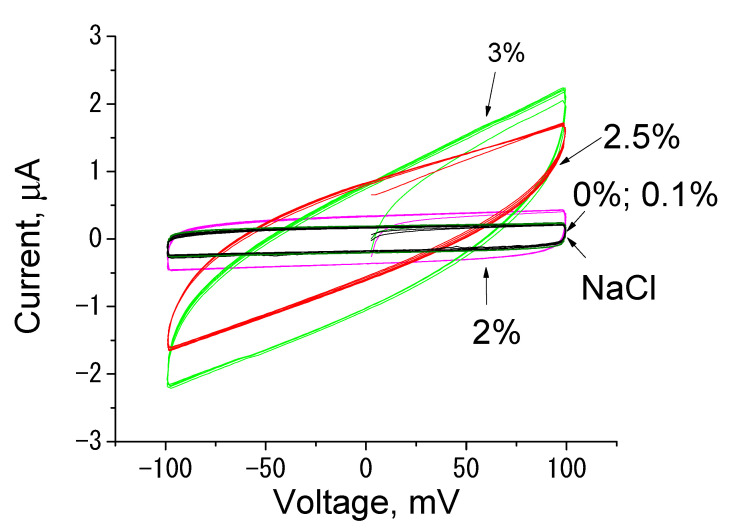
Cyclic voltammograms of chitosan/SWCNT composite films obtained in physiological solutions. The numbers denote weight percentages of SWCNT in composites. NaCl: cyclic voltammogram of electrodes of the measuring cell filled with electrolyte in the absence of the film.

The changes in the character of electric current in physiological solution are caused by the appearance of ionic charge carriers in the sample and the formation of a double electric layer in the bulk of composite film. The double electric layer is formed at the interface of an electronic conductor, in our case, the SWCNT percolation network surrounded with the chitosan scaffold. The formation of a layer of counterions on the SWCNT interface compensates the external potential; as a consequence, the current value decreases. The higher the SWCNT content, the higher the contribution of electronic current (in comparison with ionic current). In the sample containing 0.1 wt.% of SWCNT, only ionic currents are observed, as there is no SWCNT percolation, and electrical conductivity of the sample is similar to that of pure chitosan (Figure 10).

The formation of a double electric layer is confirmed by the analysis of cyclic voltammograms of the studied samples in electrolyte. The area enclosed by the curve is proportional to capacitance of the double electric layer of the sample. Figure 10 presents cyclic voltammograms of the chitosan/SWCNT films in physiological solution. The capacitance of measurement cell electrodes in physiological solution is given for comparison. It is seen that the sample containing 3.0 wt.% of SWCNT demonstrates the maximum electrochemical capacitance. Capacitance of the samples decreases with decreasing filler content. The capacitance values of the pure chitosan sample and the film with minimal content of nanotubes (0.1%) virtually coincide with that of cell electrodes in physiological solution (aqueous solution of NaCl). The results suggest that capacitance characteristics of the studied samples are determined by the formation of the percolation network of nanotubes surrounded with the hydrophilic chitosan scaffold. Chitosan, which possesses its own ionogenic groups and intercalates ions from physiological solution, creates the double electric layer on the SWCNT interface. With a decrease in nanotube content, capacitance of the sample declines. The most noticeable drop in this parameter is observed in the range of conducting component concentrations from 2.5 to 2.0 wt.%; here, the presence of the developed percolation cluster is predicted. At lower contents of SWCNT, electronic current in the film is lower than ionic currents of the electrolyte, and the capacitance component of the film does not manifest itself against the background of these ionic currents.

Note that applying cyclic potential to dry or wet composite scaffolds for 4 h causes no decrease in the current flowing in these scaffolds. In addition, electrochemical characteristics of the scaffolds are reproducible both in the dry state and in the physiological solution after prolonged storage (for 1 year) under normal conditions. This indicates stability of the studied materials and the possibility of long-term storage and reuse of the scaffolds, which are important factors in cellular therapy.

To investigate biocompatibility of chitosan/SWCNT composite films, MTT tests of the culture of human dermal fibroblasts were performed after growing cells on the composite surfaces for 4 days. It is known [30] that optical absorption of a suspension containing human fibroblasts at 570 nm is proportional to the amount of cells. Thus, it is possible to estimate the influence of composition of nanotube-containing scaffolds and alternating electric field on adhesion and proliferative activity of human fibroblasts.

Chitosan films are able to adsorb various substances and swell in solutions [31]; therefore, the possibility of application of the prepared composite films as substrates for MTT tests was investigated. Intensity of accumulation of formazan crystals in cell cytoplasm was studied using three types of substrates: a standard culture plastic, the chitosan film, and the composite film containing 2.5 wt.% of SWCNT.

**Figure 11:**
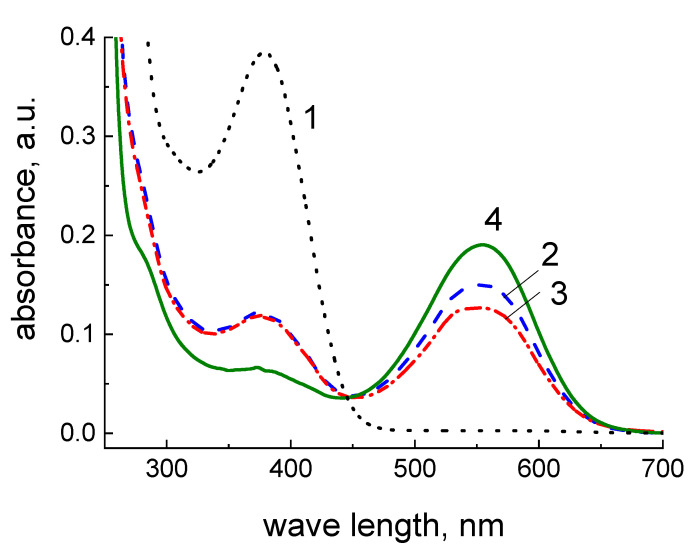
Electronic absorption spectra of MTT reagent (curve **4**) and formazan solutions in DMSO obtained after using a culture plastic (**1**), a pure chitosan film (**2**), and the composite film containing 2.5 wt.% SWCNT (**3**) as substrates.

Figure 11 presents absorption spectra of aspiration liquid on the chitosan film, the composite film containing 2.5 wt.% SWCNT, and the spectrum of the initial MTT solution. Since the absorption maxima observed upon conversion of MTT reagent into formazan on different substrates do not shift and are located at 555 nm, the wavelength of 570 nm can be used for registration of optical density and estimation of intensity of accumulation of formazan crystals when using chitosan as a substrate. The absorption peak at 378 nm present in the spectra is also observed in the spectrum of the initial MTT reagent; apparently, it is related to the presence of residual reagent in the aspiration liquid. The pure chitosan and the composite films contain higher amounts of MTT that is caused by high sorption capacity of these substrates. The intensities of absorption bands with the maximum at 378 nm are similar for pristine chitosan and the composite film; this parameter is considerably lower for the culture plastic support. Thus, due to sorption activity of the chitosan films and their tendency to swell, it is reasonable to perform comparative analysis of chitosan-based supports before the MTT test.

**Figure 12:**
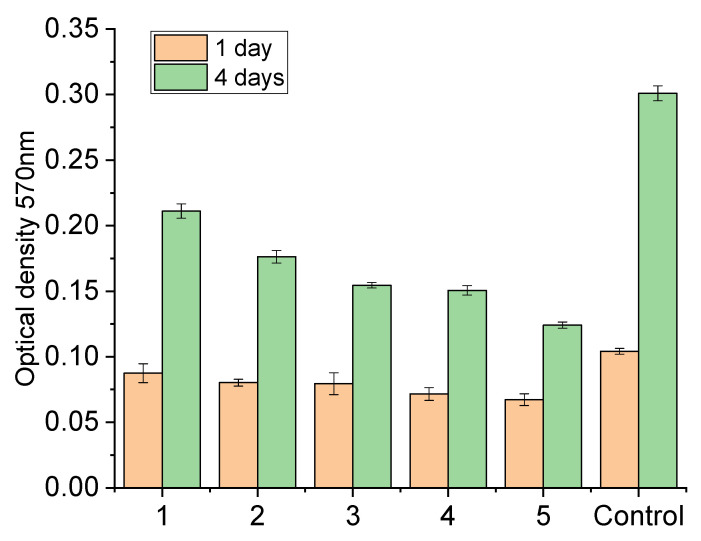
MTT tests of human dermal fibroblasts cultivated on the surface of films for 1 and 4 days: (**1**) CS, (**2**) CS-0.5 wt.% SWCNT, (**3**) CS-2 wt.% SWCNT, (**4**) CS-2.5 wt.% SWCNT, (**5**) CS-3 wt.% SWCNT. Control: the cells seeded on the surface of cultural plane (treated polystyrene surface).

Biocompatibility of the prepared materials was analyzed using the results of the MTT test obtained after cultivation of human dermal fibroblasts on the surface of the films for 1 and 4 days (Figure 12).

Introducing the amounts of the filler (SWCNT) necessary to prepare composites did not have a noticeable influence on adhesion of cells to substrates 1 day after beginning of cultivation. On the fourth day, the cells grown on the samples containing SWCNT demonstrated lower proliferative activity than those cultured on the pure chitosan film (Figure 12). This is possibly caused by changes in topography and chemical composition of the film surface: its roughness increases 10-fold (Figure 5) as compared to pure chitosan, and the maximum attributed to C-C bonds in carbon nanotubes appears in the energy region of 283.42 ± 0.2 eV (Figure 6).

## 5. Electrostimulation of Human Dermal Fibroblasts

All tissues of a living organism are electrosensitive to a greater or lesser extent. Their normal functions depend on electric signals delivered to cell membranes; these functions can be modulated via exogenous electrostimulation. Parameters of electric impulses (voltage, amperage, frequency) regulate adhesion, proliferation, and differentiation of cells of an organ or a tissue not only immediately after action, but also during the following passages. In this work, the influence of parameters of electric field on cellular processes was studied using human dermal fibroblasts.

The choice of the type of electric signal was based on the assumption that use of alternating current would help avoid negative influence of prolonged action of direct current (polarization and imbalance of electric charge, accumulation of toxic side products, and electrochemical burns) [32].

Bioelectric currents with densities varying from 0.1 μA/cm^2^ to 100 μA/cm^2^ are critical factors in launching and regulation of several important biological processes, including regeneration of damaged skin and soft tissues [33,34]. Therefore, this range of current densities, being the closest to physiological values, was selected for studying the influence of electrostimulation on cell processes. In determining physiological ranges of voltage and frequency of electric signals, the data reported in [35] were used; the authors demonstrated that the optimal parameters for skin regeneration include voltage varying from 10 to 200 mV, with the frequency not exceeding 1 Hz.

**Figure 13:**
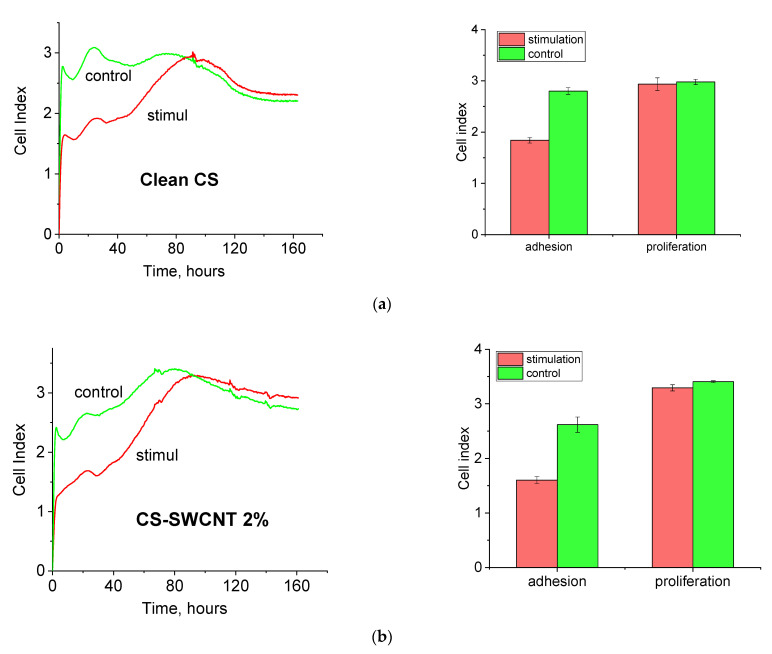

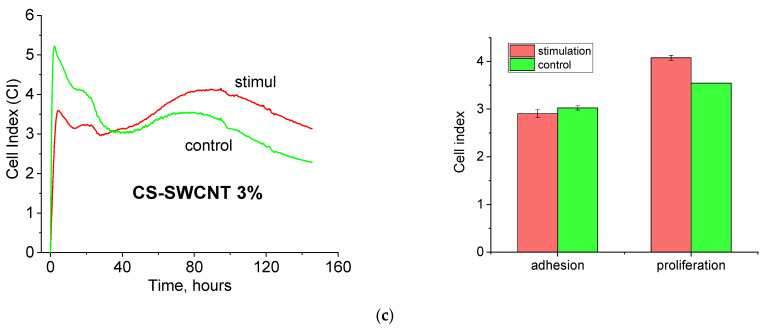
Dependences of the cell index (CI) value on the time of cultivation of human dermal fibroblasts on chitosan-based films containing 0 (**a**), 2.0 (**b**), and 3.0 *(***c***)* wt.% of SWCNT.

Figure 13 shows real time dynamics of changing cell index (CI) of the cells cultivated on RTCA iCELLIgence electronic microplates. These curves allow one to monitor dynamics of adhesion and proliferation of human dermal fibroblasts of the passages following electrical stimulation and to estimate the value of cell index at the moments corresponding to efficient cell adhesion and the maximum proliferative activity on the surface of electronic microplates.

It is clear from Figure 13a that electrostimulation of cells on the chitosan film results in a certain decrease in the level of efficient adhesion of cells of subsequent passages on electronic microplates. Even at higher proliferative activity (in comparison with that of intact cells), the maximum parameter of proliferative activity remains comparable to that of the control cells. Similar dynamics are typical of the cells stimulated on the composite film containing 2 wt.% of SWCNT (Figure 13b). The cells stimulated on the composite films containing 3 wt.% of SWCNT demonstrate a relatively low level of efficient adhesion on electronic microplates (similar to that of the control cells). Their proliferative activity is higher than that of the intact cells, and the maximum parameter of proliferative activity is significantly higher than that of control cells (Figure 13c).

One of the reasons for the observed increase in proliferative activity of cells on the scaffold with 3 wt.% SWCNT is most likely the presence of the developed percolation network of carbon nanotubes. There is no percolation network in the pure chitosan film (0% SWCNT), and in the scaffold with 2 wt.% SWCNT this network is probably insufficiently developed and cannot influence cell vital activities. Moreover, electronic currents are suppressed due to formation of the double electric layer, and the total current in the sample is only slightly higher than the currents in electrolyte (Figure 10).

The presence of the developed percolation network has a dramatic influence on distribution of currents within the scaffold. When the film without SWCNT is stimulated, no electronic conductivity is observed, and ionic currents flow mainly in the area near electrodes. Change of polarity of the electric signal leads only to redistribution of currents in the vicinity of electrodes and does not affect the whole film surface. In this case, the amount of cells subjected to the influence of the electric field is minimal. In the sample containing 2 wt.% of SWCNT with respect to chitosan, carbon nanotubes are uniformly distributed in the bulk of the chitosan scaffold, but their amount is insufficient to form the developed conducting structure. This is confirmed by low currents for the film with 2 wt.% SWCNT (Figure 9 and Figure 10), which are comparable with currents in electrolyte. As a consequence, we observe the situation described above, when electrical currents are closed in the areas near electrodes, and flow predominantly in physiological solution and not in the film. Another picture is seen in the case of the sample containing 3 wt.% of SWCNT (with respect to chitosan). The percolation network of nanotubes, which are homogeneously distributed in the chitosan scaffold, causes appearance of electronic current in the bulk of the film. Electronic current has an influence on ions localized in the near surface layer of the chitosan scaffold, which results in appearance of ionic currents. Consequently, ionic currents are no longer closed in the area around electrodes; they flow in the near surface layer of the chitosan film over the whole surface of the material. In this case, the amount of cells subjected to the influence of the electric field increases considerably, leading to the pronounced biological effect of electrical stimulation.

## 6. Conclusions

Conducting films based on chitosan and single-wall carbon nanotubes were developed. It was demonstrated that conductivity of the composite films increases from 10^−7^ to 10 S/m with increasing SWCNT content from 0.1 to 3.0 wt.%.

The films containing 0.5 wt.% of SWCNT have enhanced strength (σ = 179.4 MPa) and elastic modulus (E = 3.2 GPa) as well as high tensile strain (ε = 57.6%).

The high mechanical characteristics of the composite film with the introduction of 0.5 wt.% SWCNT are explained by the good dispersion of nanotubes in the volume of the chitosan matrix and the formation of a denser packing of chitosan macromolecules in comparison with a pure chitosan film, which is confirmed by the research results.

Analysis of XPS spectra of the composite films revealed the presence of SWCNT on their surface, which leads to an increase in surface conductivity at filler content exceeding 2.0 wt.%.

Comparative study of the conductivity of the dry films and the films immersed in physiological solution demonstrated that the chitosan scaffold filled with electrolyte forms the double electric layer at the interface of the electronic conductor, which leads to a decrease in the total conductivity of the scaffold. To maintain the currents that affect cellular material in physiological solution, the composite scaffold should contain 3 wt.% of SWCNT (in this case, the electronic component of conductivity is sufficiently high).

One of the reasons for the increase in the proliferative activity of cells for a matrix of 3 wt.% SWCNT can be considered the presence of a developed percolation network of carbon nanotubes. The percolation network of uniformly distributed nanotubes over the chitosan matrix leads to the appearance of an electron current in the volume of the entire film. The electron current affects the ions located in the surface layer of the chitosan matrix, which leads to the appearance of ion currents. The number of cells exposed to the electric field, in this case, increases significantly. This leads to a pronounced biological effect of electrical stimulation of cells.

The performed in vitro experiments demonstrated that electrostimulation of human dermal fibroblasts on the composite films for 4 h with alternating U-shaped voltage (±100 mV, polarity changing every 30 s) does not cause cytotoxicity, whereas proliferative activity of the stimulated cells increases.

## Figures and Tables

**Table 1:** Mechanical characteristics of the chitosan-based composite films containing SWCNT.

SWCNT Content, Wt. %	Tensile Strength, MPa	Young’s Modulus, GPa	Tensile Strain, %
0	124.0 ± 5.42	2.62 ± 0.57	36.68 ± 4.84
0.1	157.74 ± 15.40	3.23 ± 0.24	46.09 ± 8.13
0.5	179.40 ± 5.40	3.22 ± 0.37	57.64 ± 3.57
1.0	161.04 ± 7.98	3.35 ± 0.27	47.14 ± 2.33
3.0	158.91 ± 17.23	3.57 ± 0.26	41.16 ± 5.82

**Table 2:** Mechanical characteristics of the pure chitosan film and the composite film containing 3.0 wt.% of SWCNT measured in physiological solution.

SWCNT Content, Wt. %	Tensile Strength, MPa	Tensile Strain, %
0	32.7 ± 11.9	139.22 ± 11.22
3.0	92.93 ± 3.26	183.44 ± 15.91

## Data Availability

The data presented in this study are available on request from the corresponding author.
